# Improvement of Breast Cancer-Associated Pulmonary Tumor Thrombotic Microangiopathy With Carboplatin and Gemcitabine

**DOI:** 10.7759/cureus.38600

**Published:** 2023-05-05

**Authors:** Helen Tran, Nader Kamangar

**Affiliations:** 1 Internal Medicine, Olive View University of California Los Angeles Medical Center, Sylmar, USA; 2 Medicine, University of California Los Angeles David Geffen School of Medicine, Los Angeles, USA; 3 Medicine/Pulmonary, Critical Care and Sleep Medicine, University of California Los Angeles David Geffen School of Medicine, Los Angeles, USA; 4 Pulmonary and Critical Care Medicine, Olive View University of California Los Angeles Medical Center, Sylmar, USA

**Keywords:** respiratory failure, tumor emboli, metastatic breast cancer, pulmonary hypertension, pulmonary tumor thrombotic microangiopathy

## Abstract

We present the case of a 50-year-old woman with stage IV invasive ER+/PR-/HER2-ductal breast carcinoma who was admitted to the intensive care unit (ICU) with obstructive shock and hypoxic respiratory failure due to pulmonary tumor thrombotic microangiopathy (PTTM), which significantly improved with chemotherapy. Upon presentation, her heart rate was 145 beats/min, her blood pressure was 86/47 mmHg, her respiratory rate was 25 breaths/min, and her oxygen saturation was 80% in room air. She underwent a broad non-diagnostic infectious evaluation, received fluid resuscitation, and was placed on broad-spectrum antibiotics. Transthoracic echocardiography showed evidence of severe pulmonary hypertension with a pulmonary arterial systolic pressure (PASP) of 77 mmHg. She initially required oxygen via a high-flow nasal cannula (HFNC) at 40 liters/minute and 80% FiO2 and was subsequently placed on inhaled nitric oxide (iNO) at 40 parts per million (PPM) as well as norepinephrine and vasopressin drips for acute decompensated right heart failure. Despite her poor performance status, she was started on chemotherapy with carboplatin and gemcitabine. Over the ensuing week, she was weaned off supplemental oxygen, vasoactive agents, and iNO and discharged home. Repeat echocardiography performed 10 days after the initiation of chemotherapy demonstrated marked improvement in her pulmonary hypertension with a PASP of 34 mmHg. This case highlights the potential role of chemotherapy in altering the course of PTTM in select patients with metastatic breast cancer.

## Introduction

Pulmonary tumor thrombotic microangiopathy (PTTM) is a rare complication of malignancy thought to be due to the embolization of malignant cells into the pulmonary circulation, resulting in dyspnea, rapidly progressive pulmonary hypertension, and death from right heart failure. Due to its rapid progression and the increased risk of diagnostic procedures in this setting, PTTM is often diagnosed at post-mortem. The disease is characterized by microscopic tumor emboli and remodeling of the pulmonary vasculature, leading to acute decompensated right heart failure, acute hypoxemic respiratory failure, and, ultimately, death in a matter of days. There are no known effective treatments that reverse the histopathological changes caused by PTTM. Little is known about the optimal management of PTTM and proposed treatments rarely reverse the course of the disease. Our case illustrates the importance of establishing an early diagnosis of PTTM and the potential role of chemotherapy in altering its course in select patients with metastatic breast cancer.

## Case presentation

A 50-year-old woman with stage IV invasive ER+/PR-/HER2-ductal breast carcinoma with metastases to the bone, complicated by pancytopenia, presented to the hospital from the oncology clinic for hypoxia, hypotension, and tachycardia. She reported shortness of breath and a productive cough for several days before the presentation. In the clinic, she had a room air arterial oxygen saturation (SpO2) of 80%, a heart rate of 145 beats/min, and a blood pressure of 86/47 mmHg. Given these findings, she was sent to the emergency department (ED). She denied any fevers, chills, headaches, photophobia, chest pain, palpitations, abdominal pain, nausea, vomiting, diarrhea, dysuria, suprapubic pain, or flank pain. She did not have any sick contacts.

**Figure 1:**
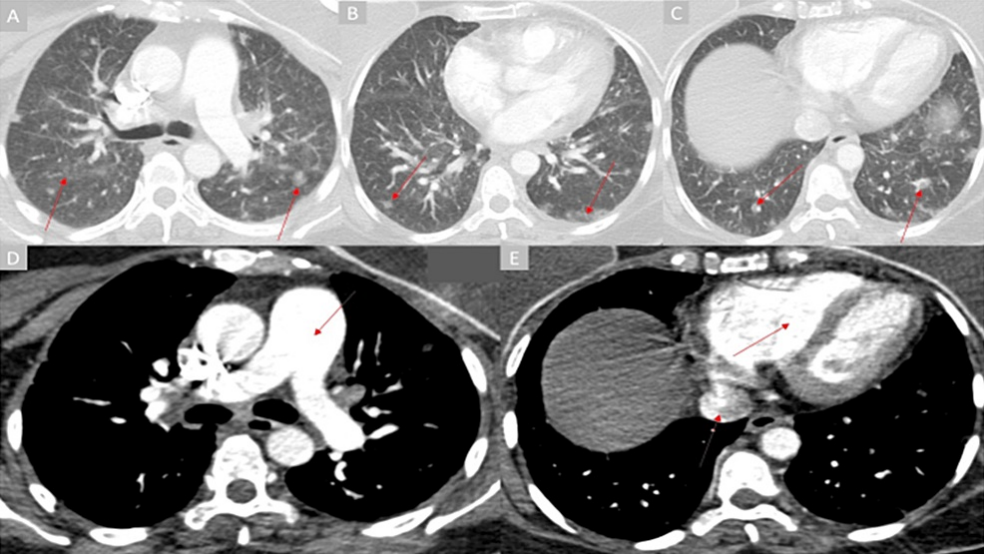
Chest CT Panels A through C: axial lung window views demonstrate the perivascular ground glass opacities and ill-defined centrilobular nodules (red arrows). Panel D: axial mediastinal window view shows an enlarged main pulmonary artery (red arrow). Panel E: axial mediastinal window view shows an enlarged right ventricle and reflux of contrast into the inferior vena cava (red arrows).

Laboratory findings were notable for a white blood cell (WBC) count of 1.6 K/cumm, hemoglobin of 10.5 g/dL, platelets of 10 K/cumm, lactate of 2.4 mmol/L, brain natriuretic peptide (BNP) of 887 pg/mL, d-dimer of 11.06 mcg/mL, and venous blood gas showing a pH of 7.44 and PaCO2 of 37 mmHg. Computed tomography (CT) pulmonary angiography was negative for acute or chronic pulmonary embolism (PE). However, she was noted to have diffuse bilateral ill-defined perivascular ground glass opacities (GGO), centrilobular ground glass micronodules, an enlarged main pulmonary artery, as well as a markedly enlarged right ventricular size with reflux of contrast into the inferior vena cava (Figure 1).

**Figure 2:**
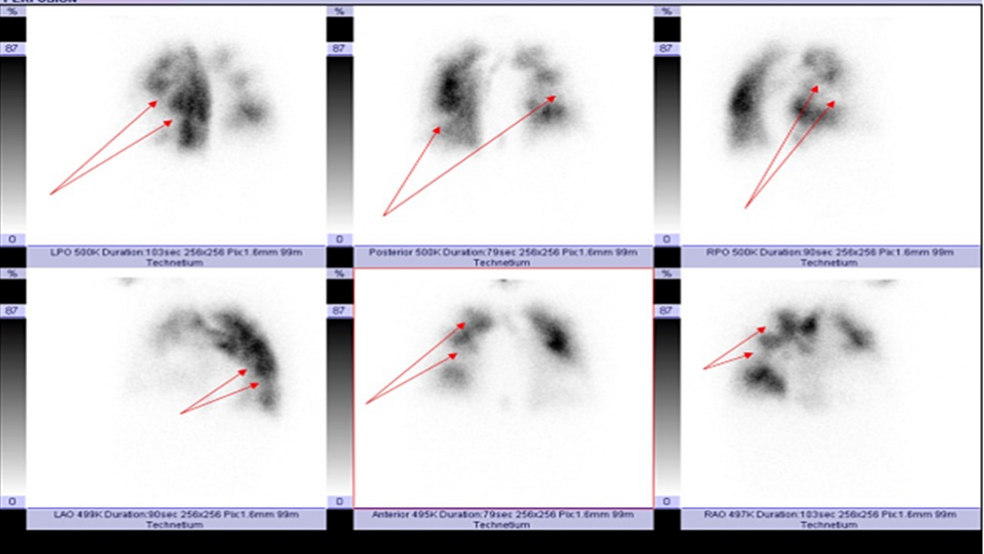
Radionucleotide perfusion scan Radionucleotide ventilation/perfusion (V/Q): perfusion images showing multiple segmental and sub-segmental perfusion defects (red arrows).

Transthoracic echocardiography (TTE) demonstrated an enlarged right ventricle (RV) with septal bowing and a pulmonary arterial systolic pressure (PASP) of 77 mmHg compared to 35 mmHg five months prior. Radionucleotide ventilation/perfusion (V/Q) scan showed multiple segmental and sub-segmental perfusion defects not evident on computed tomography pulmonary angiography with normal ventilation (Figure 2).

In addition to blood, urine, and sputum cultures, the patient had an extensive negative infectious evaluation for *Blastomycosis*, *Histoplasmosis*, *Coccidioidomycosis*, *Cryptococcus*, *Mycobacterium Tuberculosis*, *Mycobacterium avium complex*, *Pneumocystis jiroveci*, and *Legionella pneumophila*. She underwent resuscitation with intravenous fluids and was placed on broad-spectrum antibiotics and antifungal therapy, which were continued for two weeks.

The patient continued to clinically deteriorate with persistent shock and hypoxemic respiratory failure, requiring a high-flow nasal cannula (HFNC) at 40 L/min and FiO2 at 80%, as well as norepinephrine and vasopressin drip to maintain adequate mean arterial pressure. Given the TTE and the chest CT findings, as well as the negative infectious evaluation, PTTM was the suspected diagnosis. She was started on inhaled nitric oxide (iNO) as well as ambrisentan and sildenafil. Upon discussion with the oncology team and her family, it was agreed that, despite a small probability of success, chemotherapy was the only option to attempt to reverse her deteriorating clinical course. She was started on cytotoxic chemotherapy with carboplatin and gemcitabine, acknowledging the risk of treatment during a critical illness.

Over the next 14 days, her shock improved, and she was ultimately weaned off vasopressor support. Her respiratory failure also resolved, and she was weaned off supplemental oxygen. A repeat TTE done 10 days after the initiation of chemotherapy showed an improvement in her PASP to 34 mmHg, and she was subsequently discharged home. A repeat chest CT with IV contrast obtained two months after the initiation of chemotherapy showed complete resolution of the pulmonary parenchymal changes and normalization of the size of the main pulmonary artery and right ventricle.

## Discussion

Pulmonary tumor thrombotic microangiopathy (PTTM) was first described by von Herbay et al. in 1990 [1]. It is a rare complication of malignancy thought to be due to malignant cells that metastasize to the pulmonary circulation, causing remodeling of the pulmonary vasculature. This results in the rapid development of pulmonary hypertension and right heart failure, often culminating in death within weeks [1-3]. The true incidence of PTTM is unknown, although reports from autopsy cases estimate it to be between 1.4% and 3.3%, with a higher prevalence in men [1,3-5]. PTTM is most frequently associated with gastric adenocarcinoma, especially signet ring cell type, although other primary cancers, including lung, breast, ovarian, and colon, have also been implicated [1,4,5]. 

The symptom presentation of PTTM can range from weeks to months. The most commonly reported symptom of PTTM is rapidly progressive dyspnea on exertion, which often initially presents with exertion. In addition, many patients present with some degree of hypoxia, which can also rapidly progress to respiratory failure [2-4,6-9]. These symptoms are thought to be due in part to metastatic disease of the lung parenchyma, resulting in abnormalities in gas exchange and shunting. In addition, pulmonary hypertension arises from the remodeling of the pulmonary vasculature from the tumor emboli, causing an increase in right ventricular afterload and decreased right ventricular output, resulting in hypoxemia and right heart failure [2-4,6]. A dry cough is also commonly reported, and although its mechanism is not well understood, two cases from Japan reported improvement after chemotherapy [5,8].

The pathophysiology of PTTM is thought to be due to tumor emboli in the pulmonary vasculature. The cancer cells in the vasculature result in intimal damage, activation of coagulation, and recruitment of inflammatory markers [2,5-6,9]. In addition, trapped microtumors secrete local growth factors such as vascular endothelial growth factor (VEGF), tissue factor (TF), platelet-derived growth factor (PDGF), and osteopontin (OPN), which cause vascular remodeling [10-11]. VEGF, which has been reported to be upregulated by TF, is known to play a role in endothelial cell-specific angiogenesis and is hypothesized to play a role in the maladaptive changes in PTTM, as evidenced by numerous reports of PTTM biopsies that stained positive for VEGF and TF [3,10-12]. It is important to note that these maladaptive changes differentiate tumor emboli metastatic to the lung parenchyma from tumor emboli, causing PTTM. These changes ultimately result in fibrocellular intimal proliferation, deposition of platelet and fibrin microthrombi, and luminal stenosis of the pulmonary vasculature. The resulting increase in pulmonary vascular resistance causes pulmonary hypertension and can lead to symptoms previously described above, as well as rapidly progressive right heart failure and death.

PTTM is a rare entity, and therefore, the diagnosis requires a high index of suspicion in the appropriate clinical setting with supporting radiographic and histopathologic findings. Radiographic findings can be non-specific and cannot be used alone to reliably diagnose PTTM. Chest radiography can show Kerley B lines, reticular or nodular opacities, or be normal [2,4,9]. However, chest CT has been reported to show GGOs due to tumor infiltration of the alveolar septa, interlobular septal thickening due to engorgement of lymphatics, and tree-in-bud opacities [2,5,7-8]. Centrilobular tree-in-bud opacities are usually seen in infectious bronchiolitis due to inflammation and mucus production, causing dilation and plugging of small airways. However, centrilobular opacities are thought to be due to fibrocellular intimal proliferation of small pulmonary arterioles and hematogenous spread of malignancy to the pulmonary arterioles rather than plugged airways [4,13,14]. Radionucleotide V/Q scanning may show multiple small peripheral sub-segmental perfusion defects not evident on computed tomography pulmonary angiography with normal ventilation [14]. 

Histopathologic diagnosis of PTTM is often difficult as patients often rapidly deteriorate or have severe pulmonary hypertension and hypoxemia, making them unsuitable candidates for diagnostic procedures such as percutaneous, bronchoscopic, or video-assisted thoracoscopic surgery (VATS) lung biopsy. However, there have been reports of successful attempts at biopsy, including cytologic aspirate from a wedged pulmonary artery catheter, VATS, and transbronchial lung biopsy [3,15-17]. The histopathology of PTTM is characterized by tumor cells in the pulmonary vessels in addition to fibrocellular intimal proliferation and thrombosis secondary to tumor emboli in the pulmonary artery [1-2,7]. Additionally, immunohistochemical (IHC) analysis is often positive for PDGF, VEGF, OPN, and TF [2,7,11]. Right heart catheterization (RHC) may be useful in diagnosing and guiding the management of pulmonary hypertension caused by PTTM. However, management of PTTM is challenging because of the difficulty in establishing a timely antemortem diagnosis. There are no official guidelines for management, though numerous reports indicate an important factor in prolonging survival is the improvement of pulmonary pressures. Patients have been treated with chemotherapy to target the underlying tumor-causing vascular remodeling as well as targeted pulmonary vascular therapy [2,5,8,16-17]. In addition to improvement in pulmonary pressures, some patients described in these reports also had improvement in abnormalities seen on chest CT, such as interlobular septal thickening and GGOs [8]. This suggests a potential role for chemotherapy and/or targeted pulmonary vascular therapy in the management of PTTM. Further understanding of the pathophysiology of this disease is needed to generate effective management strategies.

## Conclusions

PTTM is a rare and rapidly progressive disease with a poor prognosis. Diagnosis relies on a high index of suspicion in the correct clinical setting with supportive radiographic and histopathologic findings. It should be suspected in patients with cancer, especially gastric adenocarcinoma, who present with an acute onset of respiratory failure and pulmonary hypertension in the absence of pulmonary embolism. There are no standardized treatments, though therapy aimed at reducing pulmonary pressure with chemotherapy and targeted pulmonary vascular therapy may prolong survival in some patients. Early recognition of PTTM is essential so that the initiation of diagnostic studies and potential management strategies may be employed in a timely fashion to have any potential impact on patient outcomes.
